# Supplementary material for: Non-Collagenous Dentin Protein Binding Sites Control Mineral Formation during the Biomineralisation Process in Radicular Dentin
Source: Materials (Basel). 2020 Feb 27;13(5):1053. doi: 10.3390/ma13051053 (PMC7084694; doi:10.3390/ma13051053)
Supplement: Supplementary file 1 [file materials-13-01053-s001.pdf]

materials

Supporting Information

Non-Collagenous Dentin Proteins Binding Sites Controls Mineral Formation during the Biomineralisation Process in Radicular Dentin

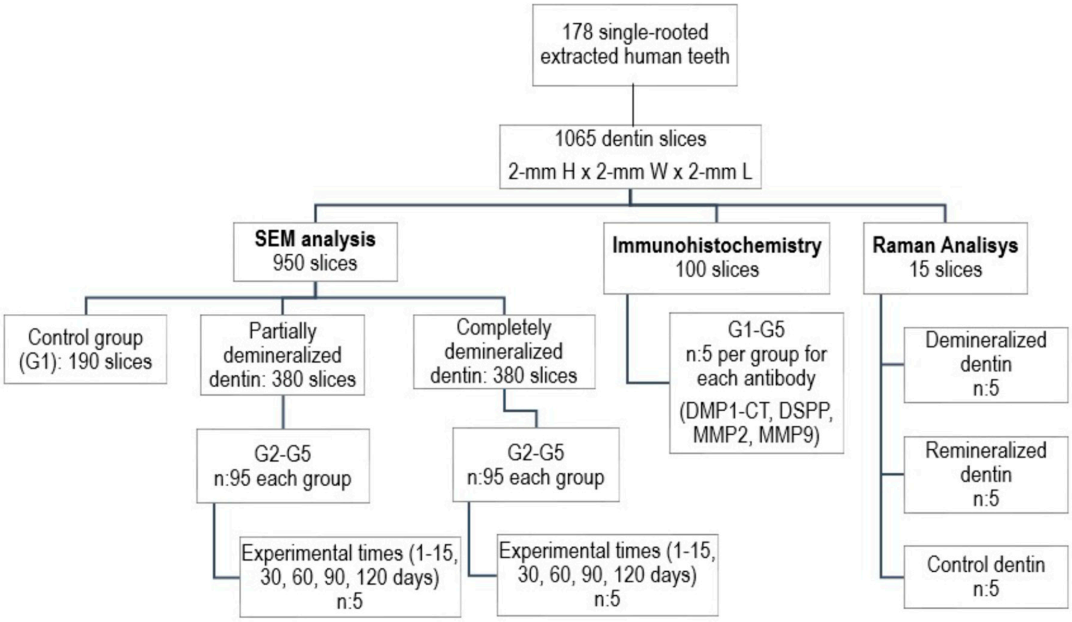

Figure S1. Flowchart of study sample selection.

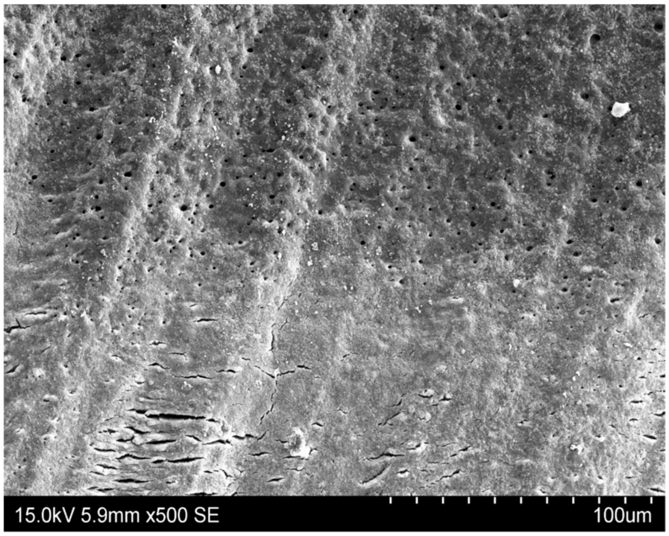

(**A**)

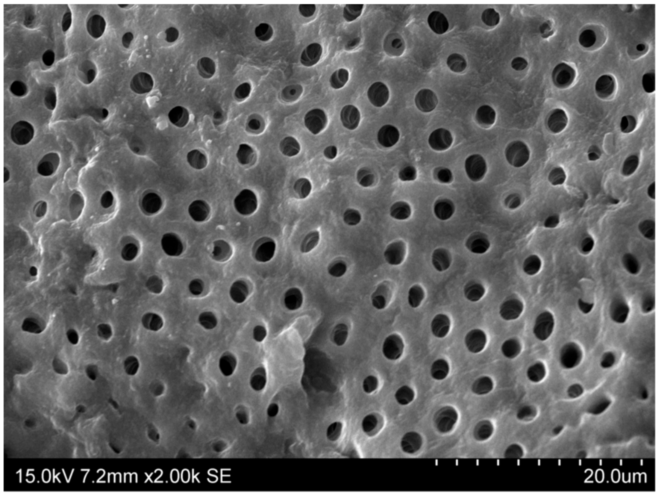
15.0kV 7.2mm x2.00k SE
20.0um

(**B**)

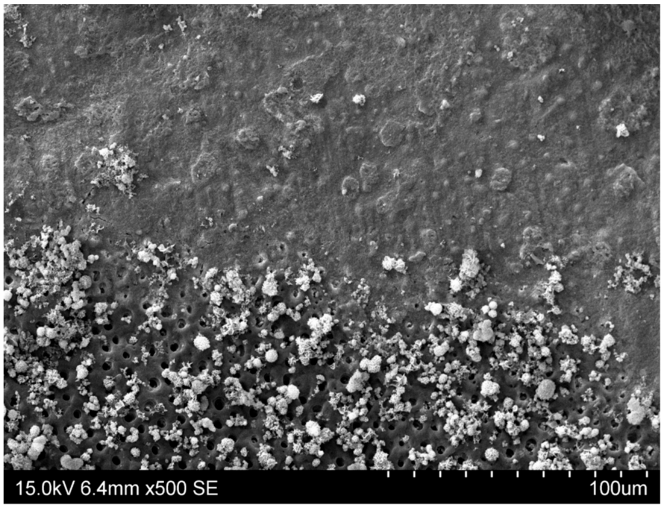
15.0kV 6.4mm x500 SE
100um

(**C**)

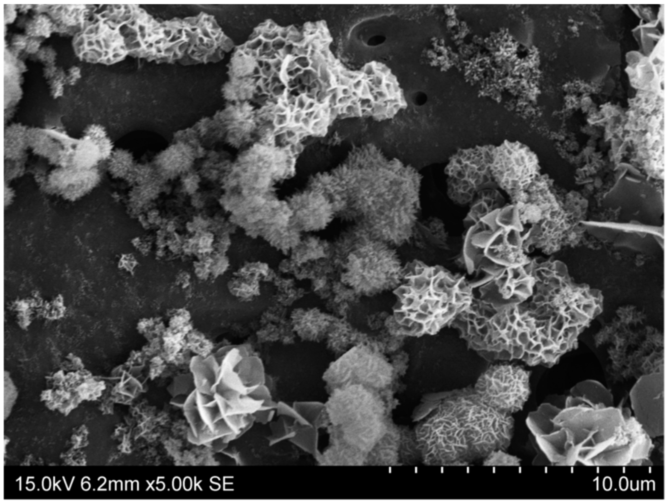
15.0kV 6.2mm x5.00k SE
10.0um

(**D**)

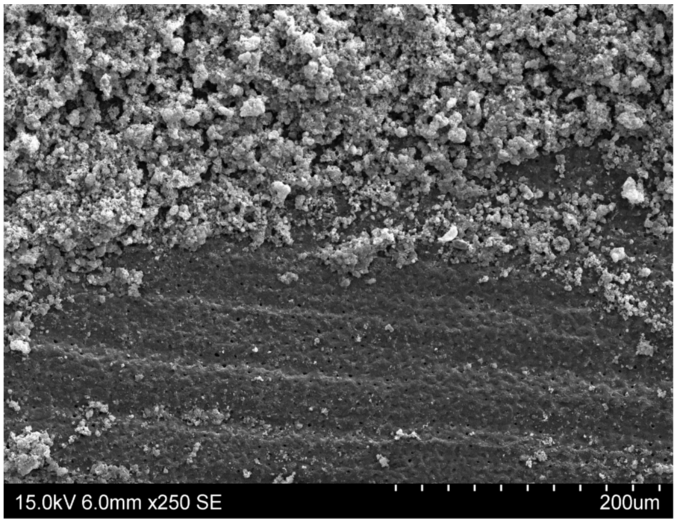
15.0kV 6.0mm x250 SE
200um

(**E**)

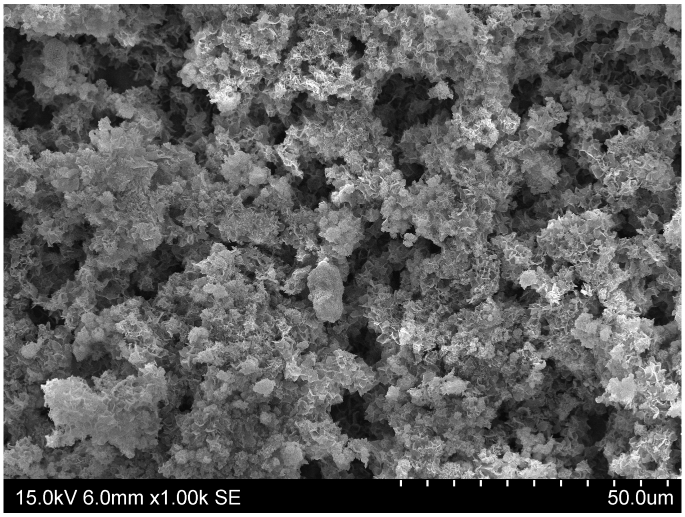
15.0kV 6.0mm x1.00k SE
50.0um

(**F**)

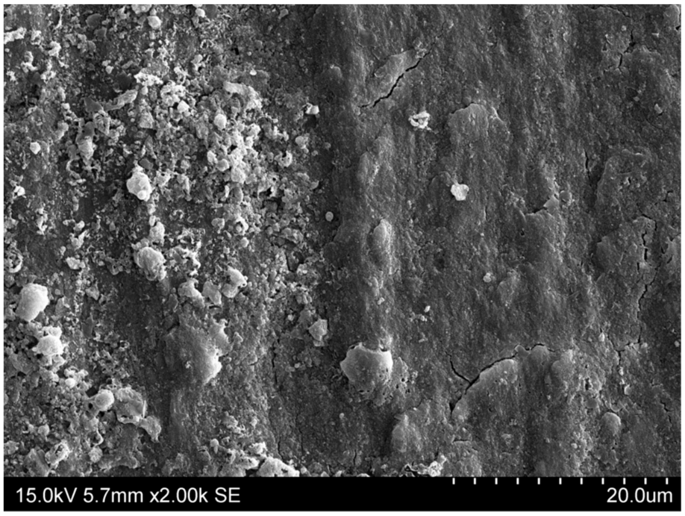
15.0kV 5.7mm x2.00k SE
20.0um

(**G**)

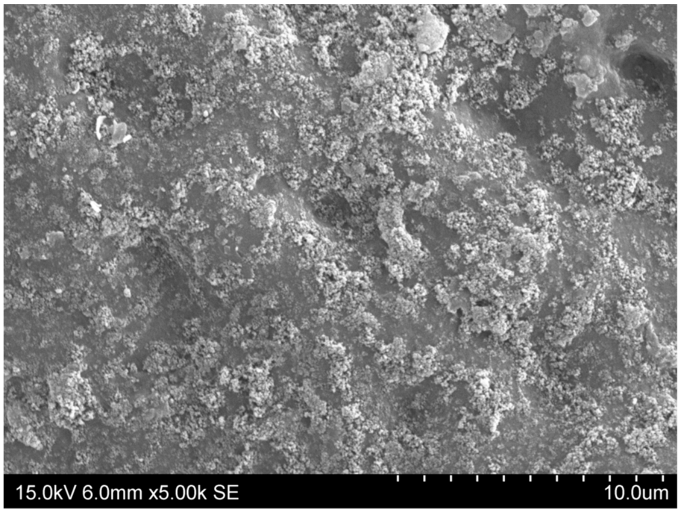

(**H**)

Figure S2. Uncropped photomicrographs showing the partially demineralised dentin as a template for remineralisation from Figure 3. To improve clarity and allow visual comparison in Figure 3, intact photomicrographs were cropped and rotated, showing control dentin at the left-hand side. Intact photomicrographs of (**A**) partially demineralised dentin (500×) and (**B**) permeable tubules (2000×); (**C**) precipitate formation exclusively over the demineralised dentin surface (500×) and (**D**) inside the intratubular dentin area (5000×); **E**(250×), **F** (2000×) thickening of the mineralised layer exclusively over the demineralized dentin at day 60; and **G** (2000X), **H** (5000×) remineralisation of radicular dentin was observed (4 months).

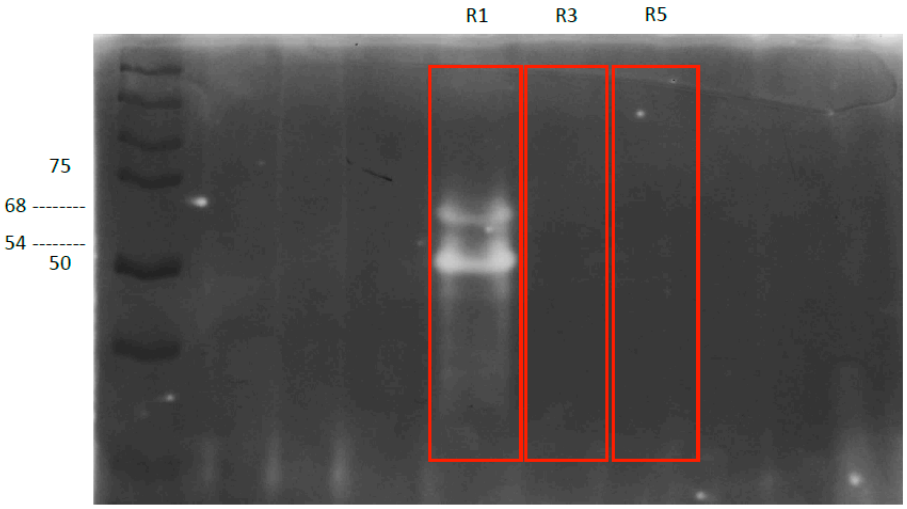

(**A**)

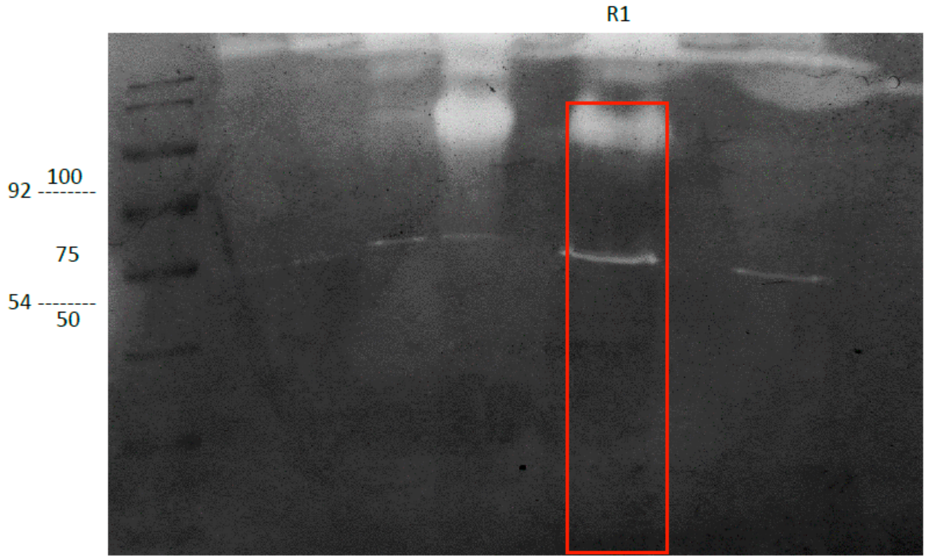

(**B**)

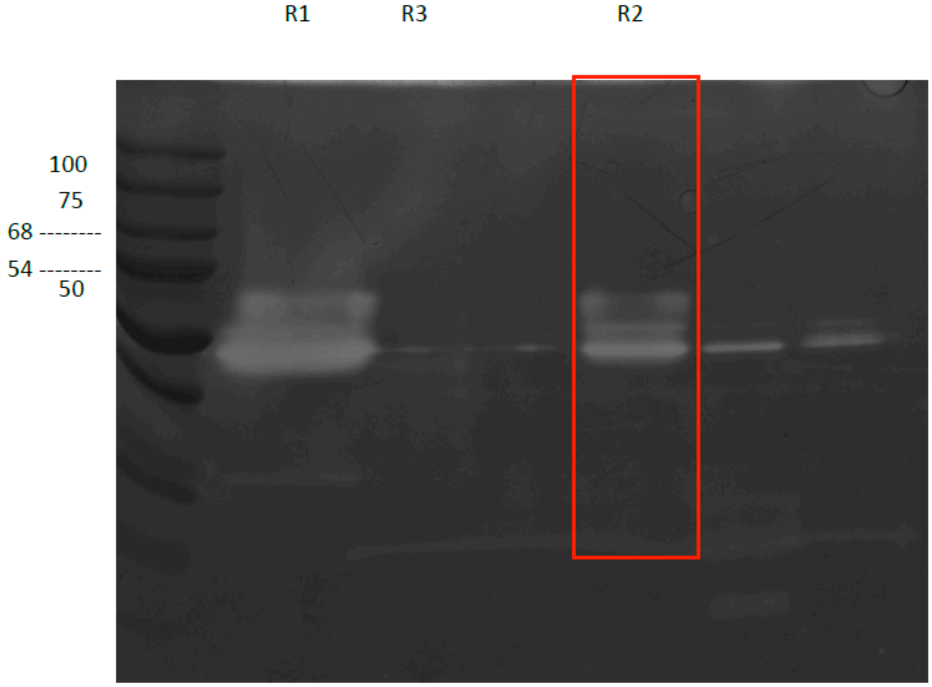

(**C**)

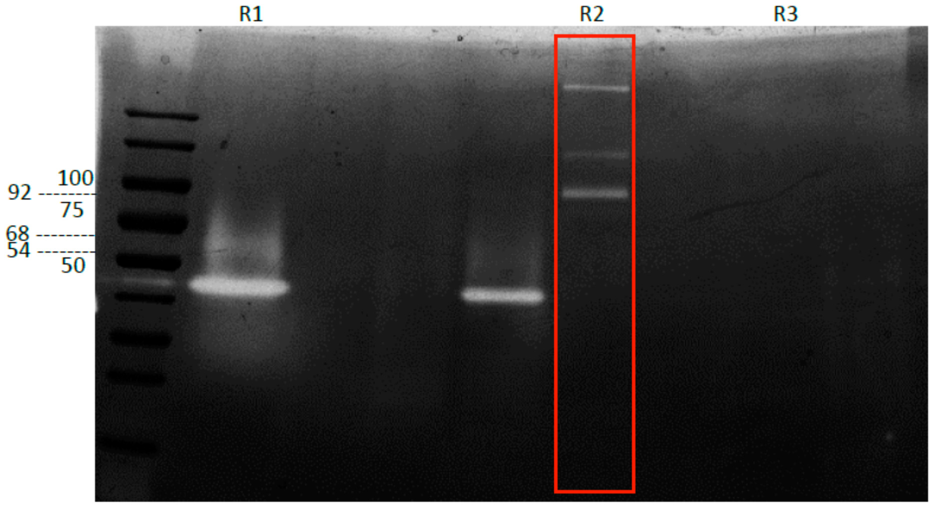

(**D**)

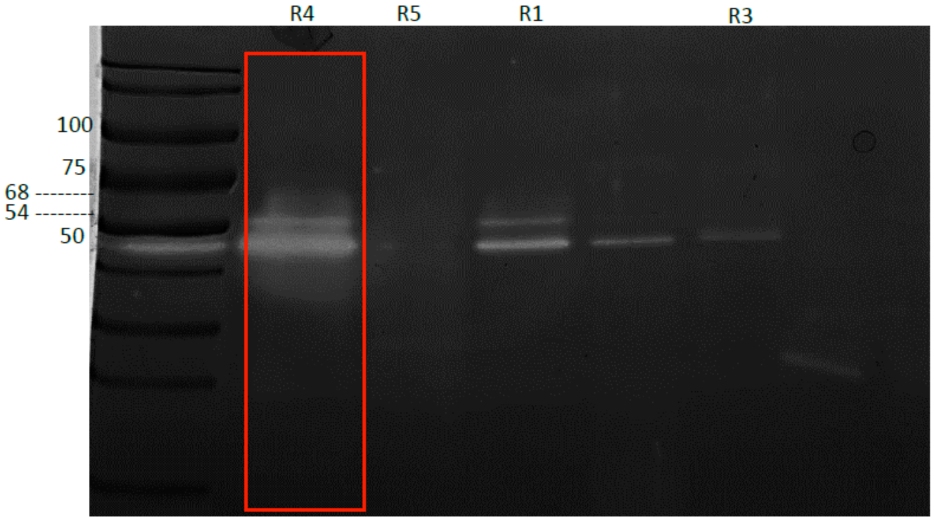

(**E**)

Figure S3. Uncropped zymograms showing the presence of MMP-2, MMP-9 and MMP-20 from Figure 4A. Different zymography gels were used to show the presence of representative gelatinolytic bands at 54 kDa (MMP-20), 68 kDa (MMP-2), and 92 kDa (MMP-9). To improves the clarity and conciseness of the Figure 4A intact zymograms from A–E were edited (brightness and contrast), cropped, rearranged and assembled. The rectangle marks the representative bands. **A**: Zymogram depicted in Figure 4A (Lane 1,5,7) show Control dentin (R1) as positive control, Demineralised dentin inhibited with chlorhexidine (R3) and Remineralised dentin inhibited with chlorhexidine (R5), both as negative controls. **B**: Zymogram depicted in Figure 4A (Lane 2) shows Control dentin (R1) at different dilutions of the sample in the uncropped gel. **C** and **D**: Zymograms depicted in Figure 4A (Lanes 3 and 4) show Demineralised dentin (R2) and positive and negative controls (R1 and R3). **E**: Zymogram depicted in Figure 4A (Lane 6), shows remineralised dentin (R4), positive (R1) and negative controls (R3 and R5).

© 2020 by the authors. Submitted for possible open access publication under the terms and conditions of the Creative Commons Attribution (CC BY) license (http://creativecommons.org/licenses/by/4.0/).
